# Smart Smile: Revolutionizing Dentistry With Artificial Intelligence

**DOI:** 10.7759/cureus.41227

**Published:** 2023-06-30

**Authors:** Ashwini Dhopte, Hiroj Bagde

**Affiliations:** 1 Department of Oral Medicine and Radiology, Rama Dental College and Research Centre, Kanpur, IND; 2 Department of Periodontology, Rama Dental College and Research Centre, Kanpur, IND

**Keywords:** patient care, treatment planning, diagnosis, dentistry, artificial intelligence

## Abstract

Artificial intelligence (AI) has emerged as a transformative technology in various industries, and its potential in dentistry is gaining significant attention. This abstract explores the future prospects of AI in dentistry, highlighting its potential to revolutionize clinical practice, improve patient outcomes, and enhance the overall efficiency of dental care. The application of AI in dentistry encompasses several key areas, including diagnosis, treatment planning, image analysis, patient management, and personalized care. AI algorithms have shown promising results in the automated detection and diagnosis of dental conditions, such as caries, periodontal diseases, and oral cancers, aiding clinicians in early intervention and improving treatment outcomes.

Furthermore, AI-powered treatment planning systems leverage machine learning techniques to analyze vast amounts of patient data, considering factors like medical history, anatomical variations, and treatment success rates. These systems provide dentists with valuable insights and support in making evidence-based treatment decisions, ultimately leading to more predictable and tailored treatment approaches. While the potential of AI in dentistry is immense, it is essential to address certain challenges, including data privacy, algorithm bias, and regulatory considerations. Collaborative efforts between dental professionals, AI experts, and policymakers are crucial to developing robust frameworks that ensure the responsible and ethical implementation of AI in dentistry. Moreover, AI-driven robotics has introduced innovative approaches to dental surgery, enabling precise and minimally invasive procedures, and ultimately reducing patient discomfort and recovery time. Virtual reality (VR) and augmented reality (AR) applications further enhance dental education and training, allowing dental professionals to refine their skills in a realistic and immersive environment.

AI holds tremendous promise in shaping the future of dentistry. Through its ability to analyze vast amounts of data, provide accurate diagnoses, facilitate treatment planning, improve image analysis, streamline patient management, and enable personalized care, AI has the potential to enhance dental practice and significantly improve patient outcomes. Embracing this technology and its future development will undoubtedly revolutionize the field of dentistry, fostering a more efficient, precise, and patient-centric approach to oral healthcare. Overall, AI represents a powerful tool that has the potential to revolutionize various aspects of society, from improving healthcare outcomes to optimizing business operations. Continued research, development, and responsible implementation of AI technologies will shape our future, unlocking new possibilities and transforming the way we live and work.

## Introduction and background

Artificial intelligence (AI) is a rapidly advancing field that encompasses the development of computer systems capable of performing tasks that typically require human intelligence. It involves the creation of computer systems and algorithms that can learn, reason, and make decisions based on data and patterns [1]. AI encompasses various subfields, including machine learning, natural language processing, computer vision, and robotics. AI has found applications in numerous industries and sectors, including healthcare, finance, transportation, manufacturing, and entertainment. Key technologies driving AI advancements include deep learning neural networks, which are capable of processing and analyzing complex patterns in data, and natural language processing algorithms, which enable machines to understand and generate human language [1].

The term "artificial intelligence" was coined by John McCarthy, a computer scientist, in 1956 during the Dartmouth Conference, where he and other researchers discussed the potential of creating machines that could simulate human intelligence [2]. Early AI research focused on solving mathematical and logical problems. Researchers developed algorithms and symbolic reasoning approaches to mimic human problem-solving abilities. In the late 1980s and 1990s, AI research shifted toward neural networks and machine learning. Researchers explored the use of interconnected artificial neurons to simulate human brain functions and enable learning from data. In the 2000s and early 2010s, the availability of vast amounts of data and advancements in computing power led to the emergence of deep learning. Deep learning architectures, particularly deep neural networks, revolutionized AI by enabling complex pattern recognition and learning from unstructured data. In recent years, AI has seen remarkable progress in several areas. Reinforcement learning, a technique where agents learn through interactions with an environment, has gained attention for its successes in game-playing AI, robotics, and optimization problems [3].

In dentistry, AI technology has emerged as a powerful tool, revolutionizing various aspects of oral healthcare. It involves the use of algorithms and machine learning techniques to analyze large volumes of data, identify patterns, and make accurate predictions. By leveraging AI, dentists can enhance diagnosis, treatment planning, patient care, and practice management [1]. In the realm of diagnosis, AI algorithms can analyze radiographs, images, and patient data to detect and diagnose oral diseases. Through machine learning, AI algorithms continually refine their diagnostic accuracy by learning from vast datasets. This not only improves the accuracy of diagnoses but also enables early detection of conditions, leading to more timely interventions and better patient outcomes [1].

AI plays a pivotal role in creating personalized treatment plans for patients. By analyzing complex patient data and considering various parameters, AI algorithms can optimize treatment plans. For example, AI can assist in the precise placement of dental implants, considering factors such as bone density, occlusal balance, and esthetic considerations. AI also enables virtual simulations and 3D modeling, providing dentists and patients with visual representations of anticipated treatment outcomes. This enhances communication and facilitates informed decision-making [3,4].

AI technology has transformed patient care in dentistry by providing personalized support and enhancing the overall dental experience. AI-powered chatbots and virtual assistants offer patients 24/7 access to information, appointment scheduling, and guidance [5]. Patients can receive answers to frequently asked questions, oral hygiene tips, and immediate support during dental emergencies. By streamlining administrative tasks, such as appointment reminders and follow-ups, AI frees up time for dental professionals to focus on patient interaction and quality care. This leads to improved patient satisfaction and enhanced overall dental experiences [6,7].

Moreover, AI has the potential to significantly enhance practice efficiency. AI algorithms can analyze patient records, treatment histories, and clinical data to identify patterns and trends. This data-driven approach helps dentists make evidence-based decisions, optimize workflows, and reduce errors. By automating routine tasks like appointment reminders and inventory management, AI technology improves productivity and reduces costs. Dentists can devote more time to complex procedures and critical decision-making, resulting in efficient practice management [7].

## Review

The integration of AI in dentistry is of paramount importance as it has the potential to revolutionize oral healthcare in several ways.

Applications of AI in diagnosis

AI has made significant advancements in the field of diagnosis in dentistry, revolutionizing the way oral diseases are detected and diagnosed. The following subsections delve into specific applications of AI in the diagnosis of oral diseases.

Dental Radiology

AI algorithms can analyze dental radiographic images, such as panoramic radiographs and cone beam computed tomography (CBCT) scans, to aid in the detection and diagnosis of various dental conditions. AI-powered image analysis can help identify and quantify dental caries (tooth decay), periodontal diseases, bone loss, and anatomical abnormalities. Automated image analysis can also assist in the detection of oral tumors, cysts, and other pathological conditions [8].

Treatment planning and implant placement: AI can aid in treatment planning for dental implants by analyzing CBCT scans. AI algorithms can simulate implant placement and provide dentists with virtual treatment plans, optimizing the placement position and angulation for optimal outcomes. For example, AI algorithms can aid in the identification of dental anomalies such as impacted teeth, supernumerary teeth, or developmental abnormalities. By comparing patient data with established norms and patterns, AI algorithms can flag any deviations and alert dentists to potential issues. AI algorithms can also assist in the detection of abnormalities in the temporomandibular joint (TMJ) and the surrounding structures. By analyzing radiographs and clinical data, AI algorithms can identify signs of TMJ disorders, such as joint degeneration, osteoarthritis, or disc displacement. This enables early intervention and appropriate treatment planning [9,10].

Image enhancement and noise reduction: AI techniques, such as deep learning, can be employed to enhance the quality of dental radiographic images by reducing noise, enhancing contrast, and improving resolution. Improved image quality can lead to better visualization of dental structures, aiding in the interpretation and diagnosis of various dental conditions. By assisting dentists in identifying suspicious areas, AI technology has the potential to improve early diagnosis and ultimately save lives [11].

Orthodontics

Cephalometric analysis and treatment planning: AI algorithms can analyze cephalometric radiographs to automatically identify and measure key anatomical landmarks used in orthodontic diagnosis and treatment planning. AI-powered software can assist orthodontists in generating cephalometric tracings, analyzing facial and dental measurements, and simulating treatment outcomes based on established treatment protocols.

Malocclusion diagnosis and classification: AI models can aid in the diagnosis and classification of malocclusions by analyzing various diagnostic data, such as dental models, facial photographs, and radiographic images. By training on large datasets, AI algorithms can learn to identify and categorize different types of malocclusions, helping orthodontists in treatment planning and determining appropriate treatment modalities. AI-powered software can generate virtual 3D models of patients' dentition and simulate the progression and outcome of orthodontic treatment. AI algorithms can automate the process of bracket placement by analyzing dental models and predicting optimal bracket positions based on individual tooth anatomy [12,13].

Periodontology

AI algorithms can analyze various diagnostic data, such as clinical parameters, radiographs, and intraoral images, to aid in the diagnosis and classification of periodontal diseases. By learning from large datasets, AI models can identify patterns and indicators of periodontal disease severity, enabling early detection and personalized treatment planning. AI-based software can automate periodontal charting by analyzing clinical data, such as probing depths, attachment levels, and bleeding scores, extracted from patient records.

Automated charting can reduce the time and effort required for manual charting, enhance accuracy, and provide a comprehensive visual representation of periodontal health status. AI-powered software can assist in treatment planning for periodontal diseases and implant placement by analyzing patient-specific data and simulating treatment outcomes. AI algorithms can aid in the selection of appropriate treatment modalities, optimize the placement and angulation of implants, and provide virtual simulations for evaluating different treatment scenarios. AI can enhance implantology procedures by assisting in implant selection, placement, and prosthetic rehabilitation. AI algorithms can analyze patient-specific anatomical data, such as CBCT scans, to optimize implant positioning, determine the appropriate implant size and type, and consider factors such as bone density and proximity to vital structures [14].

Endodontics

AI-powered software can assist in the detection and segmentation of root canals within radiographic images, helping endodontists accurately locate and analyze complex root canal systems. Automated canal detection can save time, enhance efficiency, and improve the precision of root canal treatment. AI techniques, including machine learning, can be utilized to develop predictive models that estimate the success or failure of endodontic treatments based on patient-specific data, such as clinical parameters, radiographic findings, and treatment protocols.

Instrumentation techniques: AI algorithms can analyze and optimize endodontic instrumentation techniques, such as rotary or reciprocating file systems, by considering factors such as canal morphology, anatomy, and mechanical properties. AI can also aid in optimizing irrigation protocols by analyzing the distribution and efficacy of irrigants within the root canal system, helping to improve disinfection and treatment outcomes [15].

Root fracture detection: AI algorithms can analyze radiographic images and detect root fractures, which can be challenging to diagnose clinically. Automated fracture detection can aid in identifying root fractures early, allowing for timely treatment decisions and potentially preventing tooth extraction [16].

CariScreen AI: CariScreen AI is an AI system developed by dentists and engineers that assists in the detection and diagnosis of dental caries (tooth decay). The system uses advanced algorithms to analyze dental images and provide dentists with accurate and efficient assessments of tooth decay risk. By implementing CariScreen AI, dental clinics have reported a significant improvement in the early detection of caries, leading to timely interventions and better oral health outcomes for patients [17].

Prosthodontics

AI can assist in treatment planning for prosthodontic cases by analyzing patient data, such as digital impressions, radiographs, and facial scans, to create virtual 3D models of the patient's oral anatomy. AI algorithms can aid in the design and fabrication of prosthodontic restorations, such as crowns, bridges, and dentures, by optimizing the shape, fit, and aesthetics based on patient-specific parameters and preferences.

AI-powered software can analyze digital impressions and intraoral scans to detect and delineate the margins of tooth preparations accurately. Automated margin detection can improve the precision of prosthetic restorations, facilitating optimal fit and reducing the need for manual adjustments.

Virtual articulation and occlusion analysis: AI algorithms can analyze digital models of dentition and simulate virtual articulation to assess occlusal relationships, identify interferences, and evaluate functional dynamics. Virtual articulation and occlusion analysis can aid in prosthetic treatment planning, ensuring proper occlusal alignment and harmony for optimal functional and aesthetic outcomes.

Prosthetic esthetics and shade matching: AI systems can analyze digital images of patients' natural teeth and assist in prosthetic esthetics by providing shade-matching recommendations and predicting the appearance of prosthetic restorations in different lighting conditions.

Augmented reality (AR) and guided surgery: AI-powered AR platforms can assist prosthodontists in guided implant surgery, providing real-time navigation and visualization of the implant placement process [8].

Oral Surgery

AI algorithms can analyze radiographic images, such as panoramic radiographs, CBCT scans, and MRI scans, to aid in the diagnosis of oral and maxillofacial conditions, including tumors, cysts, fractures, and impacted teeth. AI-powered software can assist in the detection, segmentation, and classification of anatomical structures and abnormalities, supporting accurate diagnosis and treatment planning. AI-based surgical planning tools can utilize patient-specific data to generate virtual surgical simulations. Surgeons can virtually plan complex procedures, such as orthognathic surgery or tumor resection, by simulating various scenarios and assessing the potential outcomes. AI algorithms can assist in surgical simulation, providing insights into the optimal surgical approach, implant positioning, and reconstruction techniques, leading to improved surgical precision and patient safety.

AI can be integrated into surgical navigation systems and robotic-assisted surgeries, enhancing the precision and accuracy of surgical interventions. AI algorithms can track anatomical landmarks, assist in real-time navigation during surgery, and provide feedback to the surgeon. This technology can be particularly valuable in complex cases involving intricate anatomical structures or challenging access, improving surgical outcomes and reducing complications. AR platforms can superimpose virtual models onto the surgical field, aiding in precise incision placement and tissue manipulation and reducing the risk of intraoperative complications [18,19].

Oral Pathology

AI algorithms can analyze histopathological images of oral tissue samples to aid in the diagnosis of oral diseases, including oral cancer, precancerous lesions, and inflammatory conditions. AI-powered systems can assist pathologists in detecting and classifying different histopathological patterns, improving diagnostic accuracy and efficiency [11].

Image analysis and feature extraction: AI techniques, such as deep learning, can be employed to analyze digital histopathological images and extract relevant features, including cellular morphology, nuclear atypia, and tissue architecture. AI algorithms can quantify these features and provide objective measurements to assist in disease diagnosis, grading, and prognosis.

Improving patient care with AI

AI has significantly improved patient care in dentistry by providing innovative solutions that enhance patient support, accessibility, and overall experience. This section delves into specific applications and benefits of AI in improving patient care.

AI-powered Chatbots and Virtual Assistants for Patient Support and Guidance

AI-powered chatbots and virtual assistants have emerged as valuable tools for patient support and guidance. These AI systems are designed to interact with patients, answer their questions, and provide assistance throughout their dental journey. Chatbots utilize natural language processing algorithms to understand and respond to patient inquiries in real-time. Patients can obtain information about dental procedures, oral health tips, post-treatment care instructions, and more. Chatbots can also guide patients in emergency situations, providing immediate support until they can receive professional care [20,21].

Virtual assistants powered by AI provide patients with personalized support and guidance. They can assist patients with appointment scheduling, reminder notifications, and follow-up care. Virtual assistants are available 24/7, allowing patients to access information and support at their convenience.

24/7 Access to Information, Appointment Scheduling, and Oral Hygiene Tips

AI technology provides patients with round-the-clock access to information, appointment scheduling, and oral hygiene tips, enhancing convenience and accessibility. Patients can access dental websites or mobile applications integrated with AI technology to obtain information about various dental procedures, treatment options, and oral health tips. AI algorithms analyze patient-specific data, such as age, medical history, and treatment preferences, to provide personalized recommendations and guidance.

Moreover, AI-powered systems enable patients to schedule appointments online without the need for phone calls or manual coordination. By integrating with dental practice management software, AI algorithms can check dentists' availability, suggest suitable time slots, and book appointments seamlessly. This eliminates wait times and simplifies the appointment scheduling process for both patients and dental practices. AI technology also offers patients personalized oral hygiene tips and reminders. Based on individual characteristics, such as age, dental conditions, and treatment history, AI algorithms provide tailored recommendations for maintaining good oral health. Patients receive reminders for routine dental check-ups, preventive care, and hygiene practices, leading to better oral health outcomes [22,23].

Enhanced Patient Experience and Satisfaction Through AI Technology

AI technology has transformed the patient experience in dentistry, leading to increased satisfaction and improved overall care. The availability of AI-powered chatbots and virtual assistants offers patients immediate support and assistance, eliminating the need to wait for responses or appointments. Patients receive prompt and accurate information, reducing anxiety and increasing trust in the dental care process.

AI-powered systems also contribute to improved patient engagement and education. By providing interactive and personalized information, patients feel more involved in their oral health journey. This empowers them to make informed decisions, comply with treatment plans, and actively participate in their dental care. The convenience and accessibility offered by AI technology enhance patient satisfaction. Patients can access information, schedule appointments, and receive support at their preferred time and location, increasing convenience and reducing barriers to care. Additionally, the proactive nature of AI-driven reminders and notifications ensures that patients stay on track with their oral health care, leading to better treatment outcomes [24,25].

Enhancing practice efficiency with AI

In recent years, AI has made significant advancements in various industries, including healthcare. AI has the potential to revolutionize medical practices by improving efficiency, accuracy, and decision-making processes. Here are some ways AI can enhance practice efficiency:

Data Analysis and Pattern Recognition for Evidence-Based Decision-Making

One of the key advantages of AI in healthcare is its ability to analyze large amounts of data quickly and accurately. Medical practices generate massive amounts of data, including patient records, test results, medical images, and research papers. AI algorithms can process and analyze this data to identify patterns, correlations, and insights that may not be apparent to human healthcare providers.

By leveraging AI, medical practices can adopt evidence-based decision-making processes. AI can analyze patient data to identify risk factors, predict disease progression, and recommend appropriate treatment options based on historical patient outcomes. This data-driven approach enables physicians to make more informed decisions, leading to better patient outcomes and reduced medical errors [26].

Workflow Optimization and Error Reduction Through AI Algorithms

AI algorithms can optimize and streamline medical workflows, reducing administrative burdens and improving overall efficiency. For example, AI-powered scheduling systems can analyze various factors such as physician availability, patient preferences, and appointment duration to automatically schedule appointments in an optimized manner. This reduces the chances of scheduling conflicts, ensures better resource utilization, and minimizes patient waiting times. Moreover, AI can assist in error reduction by identifying potential mistakes or inconsistencies in medical documentation. Natural language processing algorithms can analyze clinical notes, medical charts, and other documentation to flag errors, inconsistencies, or missing information. This helps healthcare providers identify and rectify errors before they have the potential to affect patient care [27].

Automation of Routine Tasks Like Appointment Reminders and Inventory Management

AI can automate repetitive and time-consuming tasks, allowing healthcare providers to focus more on patient care. For instance, AI-powered chatbots or virtual assistants can handle appointment scheduling, answer patient queries, and provide basic medical information. This reduces the administrative burden on staff and enhances the patient experience by providing quick and accurate responses.

AI can also automate inventory management processes, ensuring that medical supplies and equipment are available when needed. By analyzing historical usage patterns and real-time data, AI algorithms can predict inventory needs, generate purchase orders, and optimize stock levels. This eliminates the risk of stockouts, minimizes wastage, and ensures smooth operations within the practice [28].

Challenges and ethical considerations

Data Privacy and Security Concerns in AI Implementation [29,30]

Data privacy and security are significant challenges in the implementation of AI. AI systems rely on vast amounts of data, including personal and sensitive information, to train and make predictions. This creates concerns about how this data is collected, stored, and utilized. Some specific challenges and ethical considerations in this area include the following:

Informed consent: Obtaining informed consent from individuals for collecting and using their data in AI systems is essential. However, ensuring meaningful consent can be challenging, as AI often operates on complex algorithms and the implications of data usage may not be fully understood by users.

Data breaches: AI systems can be vulnerable to data breaches, which can lead to unauthorized access, manipulation, or theft of sensitive information. This raises ethical concerns about the potential harm caused to individuals whose data is compromised.

Algorithmic bias: AI algorithms can inadvertently perpetuate bias and discrimination if the training data used is biased or if the algorithms themselves are flawed. Ensuring data privacy and security involves addressing these biases to prevent unfair or harmful outcomes.

Third-party data sharing: AI implementation often involves collaborations and data sharing with third parties. Ensuring that data privacy and security standards are maintained across all entities involved becomes crucial, as any mishandling or misuse of data can have severe consequences.

Data retention and de-identification: Determining the appropriate length of time to retain data and how to effectively de-identify it poses challenges. Retaining data for too long or inadequately anonymizing it can lead to privacy breaches and compromise individual identities.

Addressing these challenges requires implementing robust data protection measures, such as encryption, access controls, and data anonymization techniques. Organizations must also adhere to relevant regulations and standards, like the General Data Protection Regulation and data protection laws specific to their jurisdiction.

Ensuring Transparency and Ethical Standards in AI Algorithms [31,32]

Transparency and ethical standards are critical for building trust in AI systems. AI algorithms often operate as black boxes, making it challenging to understand how decisions are made. Ensuring transparency and ethical standards involves the following challenges and considerations:

Explainability: AI algorithms should be able to provide clear explanations for their decisions and predictions. This is particularly important in high-stakes applications like healthcare, finance, and criminal justice, where individuals need to understand how decisions affecting them are reached.

Bias and fairness: Addressing biases in AI algorithms is essential to prevent unfair or discriminatory outcomes. Algorithmic transparency can help identify and mitigate biases by enabling scrutiny of the training data, algorithmic design, and decision-making processes.

Accountability: Establishing accountability for AI systems is crucial. Organizations must ensure that those responsible for developing and deploying AI algorithms are accountable for their decisions and actions. This includes addressing issues like algorithmic accountability, responsibility for errors or biases, and liability in case of adverse outcomes.

Ethical guidelines and frameworks: Developing and adhering to ethical guidelines and frameworks is necessary to guide the design and implementation of AI systems. These guidelines should consider factors such as human values, privacy, fairness, and societal impact.

Public trust and engagement: Ensuring transparency and ethical standards in AI algorithms requires active engagement with the public. Involving diverse stakeholders, including domain experts, policymakers, and affected communities, helps build trust and ensure that AI systems align with societal expectations.

Need for Continuous Research and Development to Refine AI Technology [33]

AI is an evolving field that requires continuous research and development to refine and improve technology. Several challenges and ethical considerations arise in this context.

Algorithmic Bias and Discrimination: As AI algorithms become more complex, addressing algorithmic bias and discrimination remains a persistent challenge. Ongoing research is necessary to develop techniques that reduce bias and ensure.

Future directions in AI dentistry

Potential Advancements in AI Algorithms and Applications [34]

The field of AI dentistry is rapidly evolving, and there are several potential advancements on the horizon in terms of algorithms and applications. These advancements aim to improve diagnosis, treatment planning, and overall patient care. Here are some key areas of development:

Predictive analytics: AI algorithms can leverage large datasets to predict disease progression, treatment outcomes, and patient-specific risks. By analyzing historical patient data, genetic information, lifestyle factors, and treatment records, AI can help dentists make more informed decisions and develop personalized treatment plans.

Natural language processing: AI systems can be trained to understand and process dental-specific terminology, patient medical histories, and clinical notes. This would enable them to extract valuable insights from unstructured data and facilitate seamless integration with electronic health records systems.

Virtual assistants: AI-powered virtual assistants can assist dental professionals in various tasks, such as appointment scheduling, patient communication, and data management. These assistants can be designed to provide evidence-based recommendations, answer patient queries, and streamline administrative workflows, freeing up more time for dentists to focus on patient care.

Integration of AI With Other Emerging Technologies (e.g., Robotics) [35]

The integration of AI with other emerging technologies holds immense potential in revolutionizing dentistry. Here are a few areas where AI can be combined with other technologies for improved outcomes:

Robotics and automation: AI can be integrated with robotic systems to enable precise and automated dental procedures. Robots can perform tasks like tooth preparation, dental implant placement, and repetitive procedures with high precision, reducing human error and enhancing treatment outcomes.

AR and VR: AI algorithms can be utilized to enhance AR and VR experiences in dental training and patient education. By merging AI-generated dental models with real-time patient data, dentists can visualize and plan treatments more accurately. Patients can also benefit from interactive simulations that help them understand procedures and visualize potential outcomes.

Internet of Things (IoT): AI can play a crucial role in analyzing data collected from connected dental devices and sensors. By processing real-time data from toothbrushes, wearables, and intraoral cameras, AI algorithms can provide personalized oral health recommendations and early detection of dental problems [36].

To further advance AI in dentistry, future research should focus on addressing the limitations identified, expanding datasets, improving data quality, and developing interpretable AI models. Deep learning algorithms, which are commonly used in AI applications, often operate as "black boxes" where it can be challenging to understand how they arrive at specific decisions or predictions. This lack of interpretability can be a concern in healthcare settings, where it is crucial to have transparency and explainability for ethical and legal reasons. Long-term studies evaluating the clinical impact of AI algorithms are needed to assess their effectiveness, safety, and cost-effectiveness. Robust studies involving diverse patient populations and comparisons with existing standards of care are necessary before widespread adoption. Additionally, ethical considerations, such as transparency, accountability, and fairness, should be integrated into the development and deployment of AI systems in dentistry. With continued research, collaboration, and careful implementation, AI has the potential to revolutionize dental practice and improve oral healthcare outcomes for patients.

Limitations of AI in Different Specialties [22]

Data availability and quality: One of the major limitations of the application of AI in dentistry is the availability and quality of data. AI algorithms require large and diverse datasets to train and validate models effectively. However, in certain dental specialties, such as rare oral diseases or specific procedures, obtaining a sufficient amount of high-quality data can be challenging. Limited data can restrict the accuracy and generalizability of AI models.

Bias and generalizability: AI algorithms are susceptible to biases present in the training data. If the training dataset is not diverse and representative of the population, the AI models may exhibit biases, leading to inaccurate predictions or recommendations. Ensuring the inclusion of diverse patient populations and data sources is crucial for reducing bias and improving the generalizability of AI models across different dental specialties.

Interpretability and explainability: AI models, particularly deep learning models, often operate as black boxes, making it difficult to interpret the reasoning behind their decisions. In dental specialties where clinical decisions have significant implications for patient care, such as oral surgery or orthodontics, it is important for clinicians to understand the factors influencing AI predictions. Lack of interpretability and explainability can hinder the acceptance and trust in AI systems by dental professionals.

Ethical and legal considerations: The use of AI in dentistry raises ethical and legal concerns. Patient privacy, confidentiality, and informed consent must be carefully addressed when collecting and utilizing patient data for AI applications. Additionally, legal frameworks and regulations regarding liability, accountability, and professional responsibility need to be established to ensure the safe and responsible use of AI in dental practice.

Clinical validation and integration: While AI algorithms show promise in various dental specialties, rigorous clinical validation studies are necessary to evaluate their effectiveness, reliability, and safety. Integration of AI systems into existing dental workflows and clinical practice may also pose challenges in terms of compatibility with existing dental software, infrastructure, and clinical protocols.

Continuous training and adaptation: AI models require regular updates and continuous training to keep up with advancements in dental knowledge and technology. Dental professionals need to stay updated with AI developments and undergo appropriate training to effectively use AI tools and interpret their outputs. Ensuring ongoing support and maintenance for AI systems is crucial for their successful implementation in different dental specialties.

Applications of Various AI Techniques in Dental Teaching [30]

Virtual simulations and training: AI can be utilized to develop virtual simulations and training modules that provide dental students with a realistic and interactive learning environment. These simulations can help students practice various dental procedures, improve their clinical skills, and enhance their decision-making abilities. AI algorithms can provide real-time feedback and guidance, allowing students to learn at their own pace and receive a personalized education.

Intelligent tutoring systems: AI-based intelligent tutoring systems can analyze student performance, identify areas of weakness, and provide customized learning materials and recommendations. These systems can adapt to individual learning styles and provide personalized feedback, allowing students to receive targeted instruction and support. AI algorithms can also assist in the assessment and grading of students' work, streamlining the evaluation process.

Adaptive learning platforms: AI can power adaptive learning platforms that tailor educational content to the specific needs and abilities of each student. These platforms can analyze student performance data, identify knowledge gaps, and deliver personalized learning materials to enhance comprehension and retention. Adaptive learning systems can also track students' progress over time, allowing educators to monitor their development and provide targeted interventions when needed.

## Conclusions

In conclusion, the future of AI in dentistry holds immense potential for advancements in algorithms and applications. Integrating AI with other emerging technologies like robotics, AR/VR, and IoT can further enhance dental care. However, addressing limitations, ensuring data privacy, and maintaining ethical considerations are crucial for the successful and responsible integration of AI in dentistry. Continued research and collaboration will be key to unlocking the full potential of AI in transforming oral healthcare. To further advance AI in dentistry, future research should focus on addressing the limitations identified, expanding datasets, improving data quality, and developing interpretable AI models. Long-term studies evaluating the clinical impact of AI algorithms are needed to assess their effectiveness, safety, and cost-effectiveness. Additionally, ethical considerations, such as transparency, accountability, and fairness, should be integrated into the development and deployment of AI systems in dentistry. With continued research, collaboration, and careful implementation, AI has the potential to revolutionize dental practice and improve oral healthcare outcomes for patients.
